# Mild encephalitis/encephalopathy with a reversible splenial lesion due to *Plasmodium falciparum* malaria: a case report

**DOI:** 10.1186/s41182-018-0119-4

**Published:** 2018-11-06

**Authors:** Momoko Mawatari, Tetsuro Kobayashi, Shinya Yamamoto, Nozomi Takeshita, Kayoko Hayakawa, Satoshi Kutsuna, Norio Ohmagari, Tomoyuki Noguchi, Yasuyuki Kato

**Affiliations:** 1 Disease Control and Prevention Center, National Center for Global Health and Medicine, Tokyo, Japan; 2 Infection Control and Prevention Center, Gunma University Hospital, 3-39-15, Showa-machi, Maebashi, Gunma 371-8511 Japan; 3 Department of Hygiene, Graduate School of Medicine, Hokkaido University, Sapporo, Japan; 4 Division of Epidemiology and Biostatistics, School of Public Health, Georgia State University, Atlanta, USA; 5 General Internal Medicine, Shonan Kamakura General Hospital, Kamakura, Japan; 6 Department of Radiology, National Center for Global Health and Medicine, Tokyo, Japan

**Keywords:** Cerebral malaria, Mild encephalitis/encephalopathy with a reversible splenial lesion, Magnetic resonance imaging

## Abstract

**Background:**

Neurological complications from malaria cause significant morbidity and mortality. Severe cerebral malaria occurs as a result of intense sequestration of infected erythrocytes in the cerebral capillaries. However, the pathology of the reversible neurological symptoms remains unclear. We report the case of a patient with malaria who also had mild encephalitis/encephalopathy with a reversible splenial lesion (MERS) causing transient neurological symptoms.

**Case presentation:**

A 55-year-old Japanese man was admitted to our hospital with acute fever upon returning from Nigeria. Blood smears and PCR analysis revealed ring forms in the erythrocytes, indicative of *Plasmodium falciparum* infection. He presented with dysarthria, expressive aphasia, and truncal ataxia, all of which were suggestive of cerebellar ataxia. He had no other signs or symptoms of severe malaria. Artemether/lumefantrine was started on the first day of illness. Although the parasites were undetectable on day 3 of illness, his neurological symptoms persisted. Brain magnetic resonance imaging (MRI) demonstrated a high-signal lesion in the splenium of the corpus callosum on diffusion-weighted images along with a decreased apparent diffusion coefficient. The neurological symptoms gradually improved by day 12. Brain MRI on day 16 showed complete regression of the splenic lesion. Therefore, the patient was diagnosed with MERS due to malaria.

**Conclusions:**

MERS often causes transient headaches, seizures, and/or impaired consciousness. The symptoms are compatible with the reversible symptoms of cerebral malaria.

## Background

Neurological complications from malaria (cerebral malaria) cause significant morbidity and mortality. Cerebral malaria occurs as a result of the intense sequestration of infected erythrocytes in cerebral capillaries, leading to brain edema and death [1]. However, some patients recover completely after they receive appropriate treatment [2, 3]. The pathology of these reversible neurological symptoms remains unclear. Although diffuse brain edema is a common finding in the magnetic resonance imaging (MRI) results of patients with cerebral malaria, there are very few case reports showing these brain MRI scans, particularly among adults [4–6]. We report the case of an adult patient with malaria who also presented transient neurological symptoms. His brain MRI revealed a reversible splenial lesion. We diagnosed this case as mild encephalitis/encephalopathy with a reversible splenial lesion (MERS) caused by *Plasmodium falciparum*. This is a rare case of a patient whose malarial neurological symptoms and the splenial lesion occurred concurrently.

### Case presentation

**Fig. 1:**
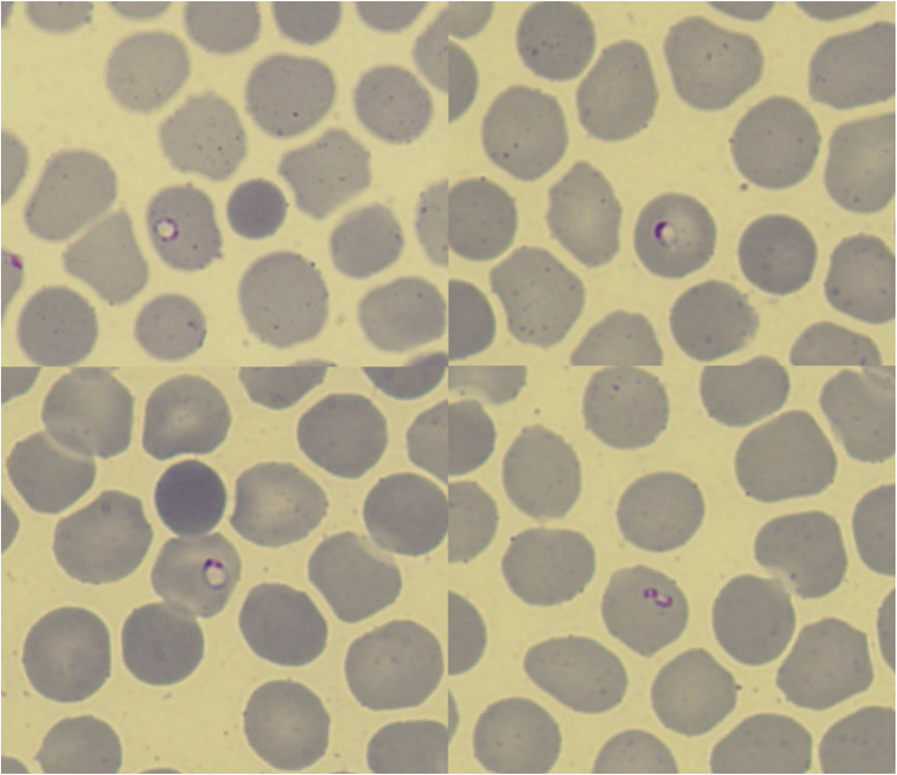
Blood smear of the patient at presentation. Photomicrograph of the blood smear shows ring forms of *Plasmodium falciparum* (× 1000 magnification; Giemsa stain)

**Fig. 2:**
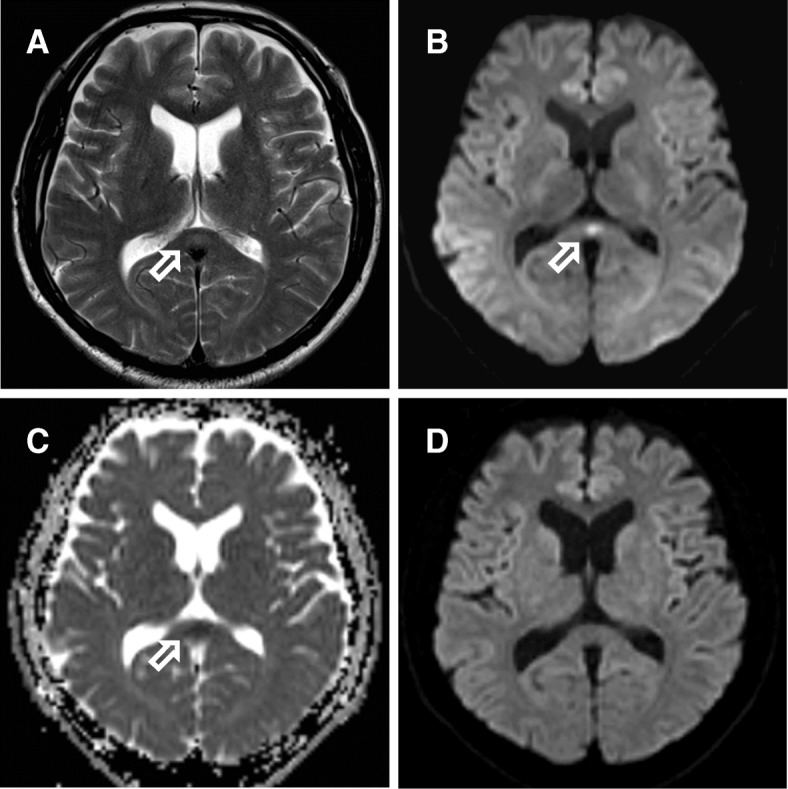
**a**–**c** Brain magnetic resonance images on day 3 of illness. **a** The T2-weighted image and **b** the diffusion-weighted image show an oval-shaped high-signal lesion in the splenium of the corpus callosum (arrow). **c** The apparent diffusion coefficient is decreased. **d** Brain magnetic resonance diffusion-weighted image on day 16 shows complete resolution of the lesion

A 55-year-old Japanese man who had returned from Nigeria was admitted to our hospital with a persistent high fever for 2 days. Blood smears revealed parasitemia with 0.05% of the erythrocytes containing ring forms (Fig. 1). The polymerase chain reaction (PCR) detected *P. falciparum*. Upon presentation, he had dysarthria, expressive aphasia, urinary incontinence, and truncal ataxia; symptoms suggestive of cerebellar ataxia. He looked dazed, and he was unable to use appropriate words in conversation. Glasgow Coma Scale score was 13 (E4V4M5). He had no other signs or symptoms of severe malaria. Hematological investigations revealed a white blood cell count of 5.8 × 10^3^ cells/μL, hemoglobin level of 18.8 g/dL, hematocrit level of 50.0%, and platelet count of 54 × 10^3^/μL. The total bilirubin, aspartate transaminase, alanine transaminase, lactate dehydrogenase, creatine kinase, serum creatinine, and C-reactive protein levels were elevated to 2.1 mg/dL, 407 IU/L, 175 IU/L, 1354 IU/L, 1593 IU/L, 1.54 mg/dL, and 6.56 mg/dL, respectively. A serum test revealed hyponatremia with a sodium level of 123 mEq/L. A computed tomography (CT) scan of the brain did not show any remarkable findings. He had no past medical history, nor had he received any vaccination in the past 5 years. On day 1 of the illness, the patient was started on oral artemether/lumefantrine treatment 3 times every 8 h at home. Since the medication was past the expiration date, we restarted another course of artemether/lumefantrine upon admission. On day 3 of the illness (day 2 of hospitalization), the parasites were undetectable by a blood test, though the neurological symptoms persisted even after the patient became afebrile on day 4. MRI of the brain demonstrated a high-signal lesion in the splenium of the corpus callosum on diffusion-weighted images with a decreased apparent diffusion coefficient (Figs. 2a–c). Analysis of the cerebrospinal fluid upon recovery of the platelet count on day 10 revealed a slightly increased total cell count (6.3 cells/μL), and normal levels of protein and glucose. While renal function recovered by day 7, neurological symptoms gradually improved by day 12, and hyponatremia improved by day 21. Brain MRI on day 16 showed complete regression of the splenic lesion (Fig. 2d). The patient was diagnosed with mild encephalitis/encephalopathy with a reversible splenial lesion (MERS) due to malaria.

## Discussion and conclusions

MERS is generally diagnosed based on radiological findings and clinical course of the disease [7]. The radiological findings are characterized by transient splenial lesions with high-signal intensity on T2-weighted images (T2WIs), fluid-attenuated inversion recovery images (FLAIR), and diffusion-weighted images (DWIs), and hyper-isointense signals on T1-weighted imaging (T1WI) sequences without contrast enhancement [7, 8]. The main symptoms of MERS include headaches, seizures, and/or impaired consciousness. Most patients recover within a month [8, 9]. Viral and bacterial infections, non-infectious diseases, or some medications have been reported to be associated with MERS. In a study of 29 pediatric MERS patients, 16 (55%) had viral diseases, and rotavirus was the most common pathogen [10]. In a study of 29 adult MERS patients, while 14 of them were not diagnosed with any disorder causing MERS, 5 had viral infections, 2 had bacterial infections, 1 had a tick bite, and 7 had other non-infectious causes [11]. Our malarial patient presented with dysarthria, expressive aphasia, urinary incontinence, and truncal ataxia, but did not have brain edema. We considered the potential association of his neurological findings with MERS. The hyponatremia observed in our patient might have contributed to developing MERS, as shown in a previous study [12], although the splenial lesion had disappeared before recovery of the serum sodium level. The clinical course and radiological findings led to the diagnosis of MERS due to *P. falciparum* infection.

Based on an extensive search, five falciparum malaria cases were with reversible splenial lesion have been reported [6, 13]; four of these were Thai adult cases. All patients were conscious and presented no neurologic abnormalities [6]. MRI examinations revealed that the splenial lesion existed on admission and improved 4 weeks after. The four patients with the splenial lesions had higher hematocrit and lower platelet count than did the patients without splenial lesions, and the hematocrit decreased more rapidly in the patients with splenial lesions. The authors mentioned that the appearance of reversible splenial lesion in malaria cases might be associated to hypoxia due to blood viscosity and hemolysis. The present patient also had a high hematocrit (50.0%) and low platelet count on admission. His hematocrit rapidly decreased to 40.0% by day 2. Therefore, high viscosity and acute hemolysis might also have occurred in our patient. Another case presenting with reversible splenial lesion was a Caucasian man living in Congo [13]; he was in a comatose state with a Glasgow Coma Scale score of 5. His first brain MRI on day 17 of illness revealed the splenial lesion. His neurological status did not improve even though his splenial lesion disappeared by day 50. He exhibited hypertension, coronary disease, diabetes, hypercholesterolemia, and nephrolithiasis—these comorbidities might have influenced his disease course. The reason the present patient presented a typical transient course of MERS might be that he had no comorbidity. However, it remains unclear whether similar reversible splenial lesions are accompanied by various neurological status in malaria patients.

Our findings suggest that MERS can be one of the clinical manifestations of cerebral malaria. It is, in fact, consistent with the reversible nature of cerebral malaria and suggests the predominance of the host immune response over the virulence of *P. falciparum* in the pathophysiology of this disease. However, accumulation and careful evaluation of MRI findings from cases of cerebral malaria are needed to confirm this conclusion.

## Abbreviations

MERS: Mild encephalitis/encephalopathy with a reversible splenial lesion
MRI: Magnetic resonance imaging
PCR: Polymerase chain reaction
